# Methanol Extracts from *Cirsium japonicum* DC. var. *australe* Kitam. and Their Active Components Reduce Intracellular Oxidative Stress in *Caenorhabditis elegans*

**DOI:** 10.3390/molecules28196923

**Published:** 2023-10-03

**Authors:** Pei-Ling Yen, Ting-An Lin, Wei Lin Chuah, Chih-Yi Chang, Yen-Hsueh Tseng, Chia-Yin Huang, Jeng-Chuann Yang, Fu-Lan Hsu, Vivian Hsiu-Chuan Liao

**Affiliations:** 1 Department of Bioenvironmental Systems Engineering, National Taiwan University, No. 1, Sec. 4, Roosevelt Rd., Taipei 106, Taiwan; d97625004@ntu.edu.tw (P.-L.Y.); r09851004@ntu.edu.tw (T.-A.L.); b09602031@ntu.edu.tw (W.L.C.); 2 Department of Forestry, National Chung Hsing University, No. 145, Xingda Rd., Taichung 402, Taiwan; a80510@gmail.com; 3 Taiwan Forestry Research Institute, No. 53, Nanhai Rd., Taipei 100, Taiwan; tseng2005@nchu.edu.tw (Y.-H.T.); yjc@tfri.gov.tw (J.-C.Y.)

**Keywords:** antioxidative activity, *Cirsium*, *Caenorhabditis elegans*, oxidative stress, silymarin

## Abstract

*Cirsium japonicum* DC. var. *australe* Kitam. has been used as an herbal remedy and often involves using the whole plant or roots. However, the bioactivities of different parts of the plant have been far less explored. This study aimed to evaluate the antioxidative ability of methanol extracts from the flowers, leaves, stems, and roots of the *Cirsium* plant and their possible active components against juglone-induced oxidative stress in the nematode *Caenorhabditis elegans*. The results showed that the highest dry weight (12.3 g per plant) was observed in leaves, which was followed by stems (8.0 g). The methanol extract yields from the flowers, leaves, and roots were all similar (13.0–13.8%), while the yield from stems was the lowest (8.6%). The analysis of the silymarin contents in the extracts indicated that the flowers, leaves, stems, and roots contained silychristin and taxifolin; however, silydianin was only found in the leaves, stems, and roots. The flower, leaf, and stem extracts, at a concentration of 10 mg/L, significantly reduced juglone-induced oxidative stress in *C. elegans*, which was potentially due to the presence of silychristin and taxifolin. Overall, *C. japonicum* DC. var. *australe* Kitam. contains a significant amount of silymarin and exhibits in vivo antioxidative activity, suggesting that the prospects for the plant in terms of health supplements or as a source of silymarin are promising.

## 1. Introduction

*Cirsium* is a genus that is categorized under the family Asteraceae and has been found in many regions, such as Asia, Europe, North America, and eastern and northern Africa [1]. In some Asian medical cultures, *Cirsium* can be used as an ingredient in traditional Chinese medicine and has been shown to cool blood, stop bleeding, and reduce liver injury [2]. Additionally, *Cirsium* extracts have been reported to have several beneficial effects; for instance, the extracts from *Cirsium rivulare* roots demonstrated in vitro antioxidative activity and exhibited antibacterial properties [3]. An in vitro study showed that the ethanol extract of *Cirsium japonicum* var. *maackii* could be utilized in the development of breast cancer treatments [4]. The methanolic extract from the aerial parts of *Cirsium japonicum* var. *ussuriense* could inhibit the production of inflammatory mediators and cytokines in cell-based experiments [5]. Although plenty of studies have reported on the pharmacological effects of *Cirsium* extracts [2], a recent study indicated that the chemical constituents in *Cirsium japonicum* extracts from different regions may vary due to the distinguishable molecular markers in the plants [6]. This suggests the importance of conducting research on native plant species to better understand their unique properties and potential benefits. Taiwanese *Cirsium* plants, with a total of eleven identified species [1,7,8], have been used as folk medicines. However, only a few studies support their medical properties; for instance, the extracts from *Cirsium arisanense* Kitam. roots and leaves reduced the hepatotoxicity induced by tacrine in Hep 3B cells and in mice [9]. *Cirsium japonicum* DC. var. *australe* Kitam. and *Cirsium kawakamii* Hayata also exerted hepatoprotective effects in mice, which may be associated with their antioxidant activities [10]. In addition, a previous study found that among seven Taiwanese *Cirsium* species, *C. japonicum* DC. var. *australe* Kitam. showed the best radical-scavenging activities in vitro [11], thereby suggesting that *C. japonicum* DC. var. *australe* Kitam. presents noticeable therapeutic potential; however, the antioxidant activities and phytochemical composition of the different parts of the *C. japonicum* DC. var. *australe* Kitam. plant remains unclear.

It has been noted that some phytochemicals identified in the *Cirsium* plants are the same as those found in silymarin, which is a well-known extract from *Silybum marianum* L. Gaertn. (milk thistle) that has been used to treat hepatic diseases [12,13]. The main phytochemicals in silymarin include silibinin, isosilibinin, silychristin, silydianin, and taxifolin [14]. Additionally, the key compounds silibinin, silychristin, and silydianin, found in silymarin, have been discovered in extracts from the *C. japonicum* DC. var. *australe* Kitam. flowers. Their antioxidant activities have also been examined in vitro and were found to be in the order of silychristin > silibinin > silydianin [11]. Another study indicated that silibinin diastereomers might contribute to the hepatoprotective effects that were observed in extracts from four Taiwanese *Cirsium* plants [10]. In addition, taxifolin is a precursor in the production of silymarin in *C. japonicum* var. *spinossimum* Kitam. [15], which implies that taxifolin may be an essential ingredient in *Cirsium* plants. Notably, taxifolin has been proven to exert anti-inflammatory [16], antioxidant [17], and hepatoprotective effects [18]. Therefore, to efficiently explore the health-promoting potential of *C. japonicum* DC. var. *australe* Kitam., an analysis of the key active constituents in different parts of the plant *C. japonicum* DC. var. *australe* Kitam. is required.

Oxidative stress, which can be caused by an imbalance between the production of reactive species and the antioxidant defense system, has been linked to inflammation and several diseases, such as cancer, diabetes, and Alzheimer’s disease [19,20]. Although oxidative stress has been receiving great attention for about 35 years, scientists continue to explore promising therapies for alleviating oxidative damage [20]. To reduce oxidative damage and improve overall health status in humans, researchers have proposed that antioxidant therapies could be a good strategy for inhibiting oxidative damage [21]. Phytochemicals are abundant in plants, and they can confer protection against environmental threats and reverse oxidative stress [22]. For example, plant-derived non-enzymatic antioxidants such as curcumin can enhance the total antioxidant capacity in humans [23]. Synthesized curcumin analogues also exhibit in vitro antioxidant activity and can reduce scopolamine-caused oxidative stress in mice [24]. Although plant-derived antioxidants have been proven to exert a wide range of bioactivities [25], the assumption that phytochemicals can alleviate oxidative stress appears to be incorrect in some clinical situations, whereby endogenous antioxidant enzymes have the potential to react faster than exogenous antioxidants [19]. Given this, some researchers have attempted to utilize phytochemicals to regulate endogenous antioxidant enzymes. Consequently, to explore the beneficial effects of plant-derived compounds, there is a need to examine whether these phytochemicals can exert antioxidant activities in organisms. 

The nematode *Caenorhabditis elegans* is widely used as an in vivo model organism. With a short life cycle, *C. elegans* takes only 3 days to grow from larvae into gravid adults. Moreover, *C. elegans* has a simple but well-investigated cell lineage [26]. The genome of *C. elegans* is highly homologous to humans, which makes it a versatile model for medical and biological studies [27]. Furthermore, *C. elegans* is a simplified and tractable system for studying the genetic and molecular aspects of stress responses; therefore, it has been used as a model organism to study stress response [28]. The highly conserved detoxification mechanisms involved in oxidative stress in *C. elegans* include the insulin signaling pathway, superoxide dismutase, and catalase [28,29]. Therefore, *C. elegans* also allows researchers to evaluate the protective effects of natural compounds through specific assays, such as oxidative stress resistance assay, and measurement of reactive oxygen species (ROS) levels [30]. In addition to aiding the exploration of the potential antioxidative activity of natural compounds, *C. elegans* can provide valuable insights into molecular and cellular information regarding the mechanisms of antioxidant activities exerted by natural compounds [30]. 

Herein, this study aimed to use *C. elegans* as a model to evaluate the antioxidative properties of methanol extracts from the flowers, leaves, stems, and roots of *C. japonicum* DC. var. australe Kitam., as well as the possible active components, silychristin and taxifolin, and silydianin during juglone-induced oxidative stress. Juglone (5-hydroxyl-1,4-naphthoquinone) is a natural toxin that induces cytotoxic effects, including ROS production, apoptosis, and DNA damage [31]; therefore, juglone has been used as a ROS-generating compound in *C. elegans* [32].

## 2. Results

### 2.1. C. japonicum DC. var. australe Kitam. Leaves Exhibit the Highest Dry Weight and Yield in Methanol Extracts

**Figure 1:**
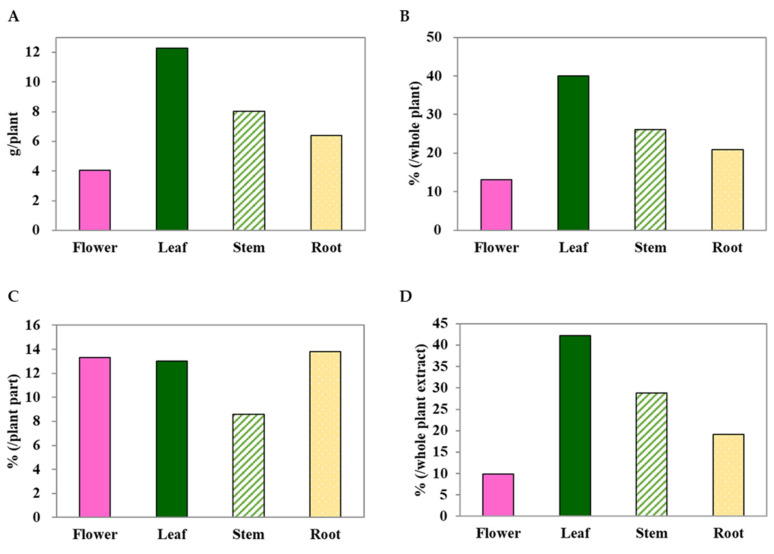
The dry weights (**A**,**B**) and yields of the methanol extracts (**C**,**D**) from *Cirsium japonicum* DC. var. *australe* Kitam. flowers, leaves, stem, and roots.

To screen the antioxidant potential of different parts of the *C. japonicum* DC. var. *australe* Kitam. plant, it was divided into four parts: flowers, leaves, stem, and roots. The results showed that the leaves exhibited the highest dry weight (12.3 g) per plant, followed by the stem (8.0 g), roots (6.4 g), and flowers (4.0 g) (Figure 1A). Similarly, the leaf ranked highest in terms of maximum dry weight percentage (39.9%), while the flowers had the lowest (13.1%) (Figure 1B). Methanol extraction was performed on these plant parts to obtain crude extracts. The results showed that similar yields of crude extracts were obtained from the flowers (13.3%), leaves (13.0%), and roots (13.8%), while the stem provided the lowest yield (8.6%) (Figure 1C). When considering the overall crude methanol extract yield, the highest crude methanol extract yield was found in the leaves, compared with the whole plant extract, followed by the stem, roots, and flowers (Figure 1D). 

### 2.2. Crude Methanol Extracts Enhance Oxidative Resistance in C. elegans

**Figure 2:**
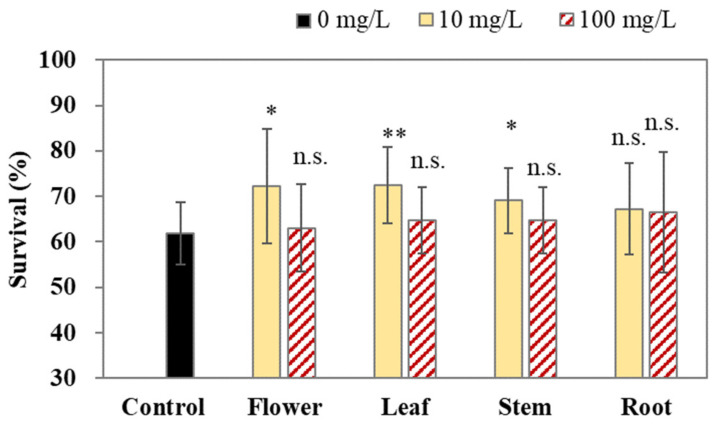
Antioxidative effects of methanol extracts from *Cirsium japonicum* DC. var. *australe* Kitam. flowers, leaves, stem, and roots in *C. elegans*. L1 larvae were treated with crude methanol extracts (0, 10, and 100 mg/L) from *C. japonicum* DC. var. *australe* Kitam. flowers, leaves, stem, and roots for 72 h, followed by the 250 μM juglone challenge for 3.5 h; the survival of the worms was scored thereafter. The assay was performed for at least 3 biological trials and at least 60 worms were scored in each trial. Data are presented as the mean ± SD, and the results from the statistical analysis are labeled with an asterisk (*p* < 0.05), two asterisks (*p* < 0.01), or n.s. (*p* > 0.05).

Next, the antioxidative activity of the crude methanol extract from the flowers, leaves, stem, and roots of *C. japonicum* DC. var. *australe* Kitam. was examined. In our study, a juglone-induced oxidative stress assay was applied to examine the oxidative resistance in *C. elegans*. Figure 2 shows that 10 mg/L of flower, leaf, and stem extracts increased the survival of the worms following exposure to 250 μM juglone for 3.5 h. Conversely, 10 mg/L of root extract did not show significant antioxidative activity (Figure 2). Moreover, 100 mg/L of the flower, leaf, stem, and root crude extracts also did not significantly increase the survival of the worms (Figure 2), thereby suggesting that a high concentration of the crude extracts could not provide beneficial effects and might exert toxic effects. Therefore, 10 mg/L of crude methanol extracts may alleviate the ROS production in *C. elegans*.

### 2.3. Development of a Chromatographic Method for the Analysis of Five Target Phytochemicals in the Methanol Extracts

**Figure 3:**
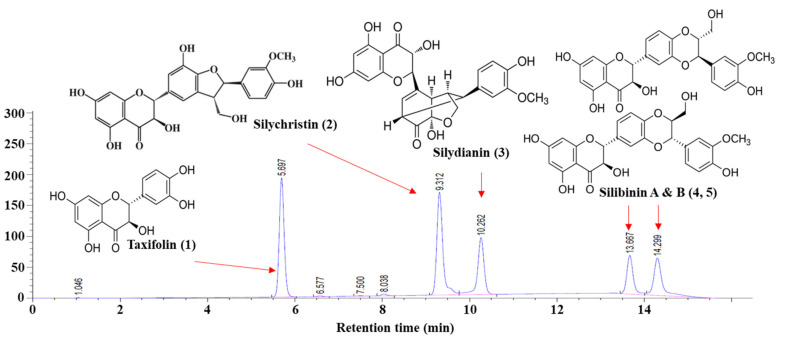
HPLC chromatogram and chemical structures of major phytochemical compounds. The concentration of each compound was 400 mg/L.

It has been reported that flavonoglignan (including silibinin, silychristin, and silydianin) and flavonoid (such as taxifolin) are major phytochemical compounds in *Cirsium* plants [11,15]. To further investigate these compounds, the chromatographic separation of a mixture of compounds (silibinin A and B, silychristin, silydianin, and taxifolin) was conducted using the HPLC-PDA system. Based on the HPLC chromatogram peak retention times, the standard compounds could be well separated, and these peaks were identified as follows: taxifolin (peak 1), silychristin (peak 2), silydianin (peak 3), silibinin A, and silibinin B (peak 4 and peak 5) (Figure 3), revealing that the chromatographic method can be used for rapidly screening target phytochemicals in *Cirsium* plants. Since the established method was suitable for separating all the target compounds, the qualitative and quantitative analyses of the target compounds in the methanolic extracts were analyzed under the same conditions.

### 2.4. Flower, Leaf, Stem, and Root Methanol Extracts from C. japonicum DC. var. australe Kitam. Contain Rich Contents of Silymarin

**Figure 4:**
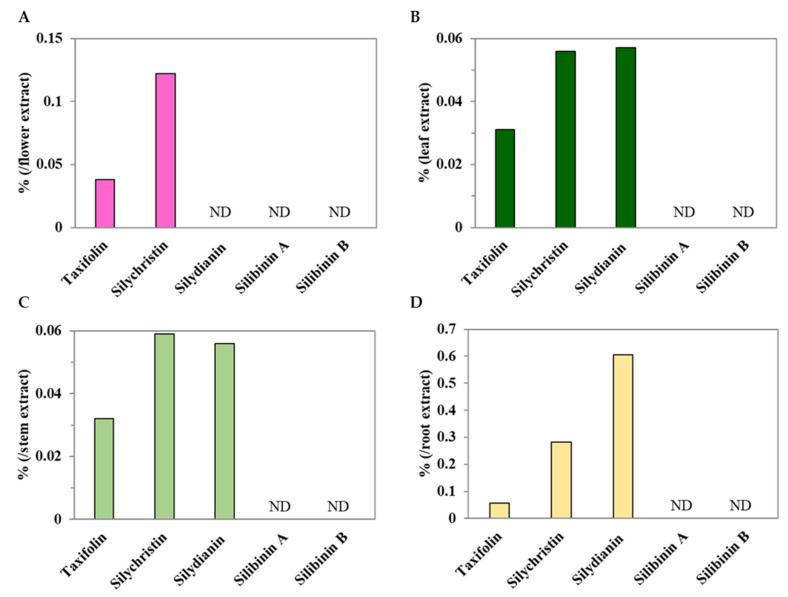
Major phytochemical compounds in methanol extracts from *Cirsium japonicum* DC. var. *australe* Kitam. flowers, leaves, stem, and roots. Major phytochemical compounds in methanol extracts from (**A**) flowers, (**B**) leaves, (**C**) stem, and (**D**) roots. ND: not detectable.

Silymarin is an extract from milk thistle (*S. marianum* L. Gaertn.) and contains flavonolignans (silybins A and B, isosilybins A and B, silychristin, isosilychristin, and silydianin) and a flavonoid (taxifolin) [33]. In this study, taxifolin and silychristin were found in the flowers, leaves, stem, and roots of *C. japonicum* DC. var. *australe* Kitam.; however, silydianin was only detected in the leaf, stem, and root extracts (Figure 4). Silibinin A and B were completely absent in *C. japonicum* DC. var. *australe* Kitam. (Figure 4). Meanwhile, the root contained the highest amount of taxifolin, silychristin, and silydianin, while the flower was second for silychristin abundance (Figure 4). Moreover, the levels of taxifolin, silychristin, and silydianin in the leaves were almost identical to those in the stem (Figure 4B,C). Thus, the results suggest that the flowers, leaves, stem, and roots of *C. japonicum* DC. var. *australe* Kitam. hold potential as rich sources of silymarin, while the root contained the highest amount of silymarin.

### 2.5. Silychristin and Taxifolin May Contribute to the Antioxidative Activity of Crude Methanol Extracts in C. elegans

**Figure 5:**
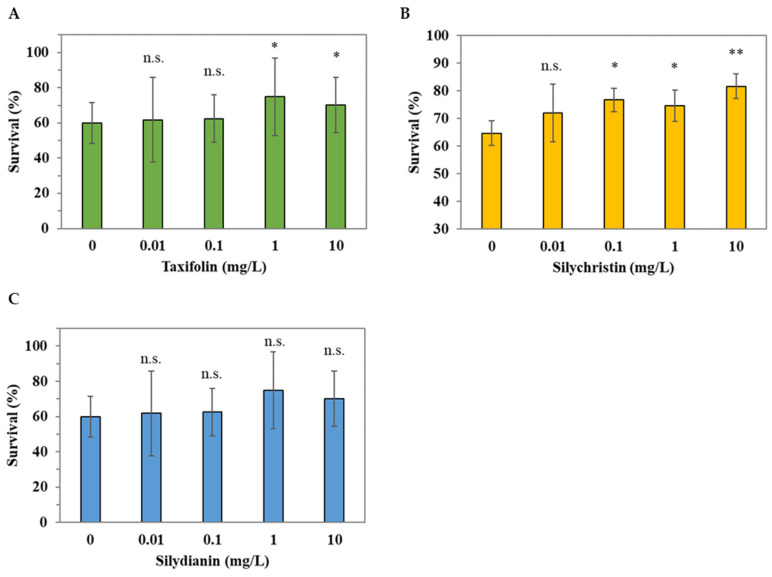
Antioxidative effects of taxifolin, silychristin, and silydianin in *C. elegans*. L1 larvae were treated with different concentrations (0, 0.01, 0.1, 1, and 10 mg/L) of (**A**) taxifolin, (**B**) silychristin, and (**C**) silydianin for 72 h followed by the 250 μM juglone challenge for 3.5 h; the survival of worms was scored thereafter. The assay was performed for at least 3 biological trials and at least 60 worms were scored in each trial. Data are presented as the mean ± SD, and the results from the statistical analysis are labeled with an asterisk (*p* < 0.05), two asterisks (*p* < 0.01), or n.s. (*p* > 0.05).

We further investigated the potential key phytochemical compounds to which the antioxidative activity observed in *C. elegans* might be attributed by analyzing the crude methanol extracts (Figure 2). Based on the results observed in Figure 4, showing that silibinin A and silibinin B were absent in the crude extracts of *C. japonicum* DC. var. *australe* Kitam., we tested the antioxidative activity of taxifolin, silychristin, and silydianin under juglone-induced oxidative stress in *C. elegans*. Figure 5A showed that 1 mg/L and 10 mg/L of taxifolin increased the survival of *C. elegans*. Moreover, silychristin exhibited the lowest observed effect level (LOEL) of 0.1 mg/L, which enhanced the survival of *C. elegans* (Figure 5B). In contrast, silydianin did not show oxidative resistance in *C. elegans* at any of the examined concentrations (Figure 5C). This suggests that silychristin and taxifolin may contribute to the antioxidative activity, derived from the *C. japonicum* DC. var. *australe* Kitam. crude methanol extracts, in *C. elegans*.

## 3. Discussion

The extraction yield of a crude extract is fundamental for screening bioactive compounds and for drug discovery. This study showed that the crude extracts from the *C. japonicum* DC. var. *australe* Kitam. flowers, leaves, stem, and roots all yielded about 13% (Figure 1), which is a similar value to the one presented in a previous study, where a yield of 10% was extracted from the flowers of *C. japonicum* DC. var. *australe* Kitam. [10]. To compare yields among different *Cirsium* species, we also calculated the crude extract yield for the aerial part of *C. japonicum* DC. var. *australe* Kitam., which amounted to 11.0%. It has been reported that the methanolic extract yields from the aerial parts of *C. arisanense* and *C. kawakamii* were 23.9% and 4.5%, respectively [10]. Therefore, in comparison to *C. arisanense* and *C. kawakamii*, the yield from *C. japonicum* DC. var. *australe* Kitam. in this study was deemed moderate. This information provides valuable insights into the extraction efficiency of *C. japonicum* DC. var. *australe* Kitam. in relation to other *Cirsium* species for further bioactive compound exploration.

Next, we investigated the antioxidative potential of the crude methanol extracts obtained from various parts of *C. japonicum* DC. var. *australe* Kitam. The extracts from the flowers, leaves, and stem led to a notable increase in the survival of *C. elegans* upon juglone exposure for 3.5 h (Figure 2). The result aligns with a previous study [11] that reported *C. japonicum* DC. var. *australe* Kitam. as having the highest contents of total phenolic compounds and the best in vitro radical-scavenging capacity among seven Taiwanese *Cirsium* species. In comparison to the aerial parts of *C. japonicum* DC. var. *australe* Kitam., both our study and the aforementioned research [11] indicate that the roots exhibit the lowest antioxidant potential. These observations shed light on the inherent variations in antioxidative properties within different plant components of *C. japonicum* DC. var. *australe* Kitam. and suggest the most suitable plant parts for future antioxidant-related investigations.

This study also examined characteristic phytochemical compounds akin to silymarin in each of these methanolic extracts. We found that the order of the highest silymarin contents was found to be root > flower = leaf = stem (Figure 4). This result suggests that the root is the best source from which to obtain silymarin compounds. Interestingly, silibinin A and silibinin B, which are the major compounds in silymarin from milk thistle, were not found in any of the crude methanol extracts in this study (Figure 4), which is different to previous studies [10,11]. This suggests that silibinin may not be the key component in the crude methanol extracts from *C. japonicum* DC. var. *australe* Kitam. In addition, in comparison with the *C. japonicum* DC. var. *australe* Kitam. flower extracts [10,11], our study provided further information on the contents of taxifolin in crude methanol extracts from *C. japonicum* DC. var. *australe* Kitam., as our results revealed that taxifolin was abundant in the roots and the aerial parts of the *Cirsium* plant (Figure 3). This information is valuable for future extraction processes aimed at harnessing the phytochemical potential of these plant components.

We investigated the antioxidative effect of silymarin in vivo and found that silychristin and taxifolin were potentially the main antioxidants in the crude methanol extracts (Figure 5). Previous studies have reported that taxifolin has several pharmacological activities, including antioxidant, anti-inflammatory, hepatoprotective, and antihyperglycemic properties [34,35]. Taxifolin may inhibit ROS generation and the induction of cell apoptosis by H_2_O_2_, which is associated with Nrf2 translocation [17]. Another study found that taxifolin may scavenge ROS and repress the genes involved in the apoptotic pathway [36]. The antioxidant activity of taxifolin may result from its structure, which consists of two phenolic groups [37]. As for silychristin, previous research has shown that silychristin may inhibit α-glucosidase activity and protect pancreatic β cells from apoptosis [38]. Moreover, silychristin may suppress apoptosis via the Nrf2 pathway in GLUTag cells [39]. The antioxidant capacity of silychristin may further increase multidrug resistance by inhibiting ABC transporters [40]. Overall, our study revealed that *C. japonicum* DC. var. *australe* Kitam. contains characteristic silymarin compounds in the root, flower, leaf, and stem, while the root is the best source from which to obtain silymarin compounds. Furthermore, our study identified that silychristin and taxifolin played a crucial role in the plant’s antioxidative ability in vivo. This study suggests that *C. japonicum* DC. var. *australe* Kitam. could be a rich source of silymarin and has the potential to be applied within the medical field or health supplements.

In this study, we explored the potential application of *C. japonicum* DC. var. *australe* Kitam. and found that the plant extracts and some ingredients exhibited health-promoting potential. It is important to acknowledge, however, that our study has certain limitations. For instance, we did not include an optimization of the processes and preparations for these plant extracts. Although the solvent was chosen according to previous research [10,11], the residual methanol may pose a health concern in the medical application of the extracts. In future research, there is an opportunity to enhance our processes by drawing on insights from successful studies related to milk thistle or silymarin [41,42], which have demonstrated effective optimization strategies. Additionally, we should note that our study did not examine the biological activity or toxicity of other ingredients; therefore, future studies should evaluate the antioxidant potential of these additional components. 

## 4. Materials and Methods

### 4.1. Chemicals

All chemical standards, including (2*R*,3*R*)-2-(3,4-dihydroxyphenyl)-3,5,7-trihydroxy-2,3-dihydrochromen-4-one (taxifolin), (2*R*,3*R*)-3,5,7-trihydroxy-2-[(2*R*,3*S*)-7-hydroxy-2-(4-hydroxy-3-methoxyphenyl)-3-(hydroxymethyl)-2,3-dihydro-1-benzofuran-5-yl]-2,3-dihydrochromen-4-one (silychristin), (1*R*,3*R*,6R,7*R*,10*R*)-3-hydroxy-10-(4-hydroxy-3-methoxyphenyl)-8-[(2*R*,3*R*)-3,5,7-trihydroxy-4-oxo-2,3-dihydrochromen-2-yl]-4-oxatricyclo[4.3.1.03,7]dec-8-en-2-one (silydianin), (2*R*,3*R*)-3,5,7-trihydroxy-2-[(2*R*,3*R*)-3-(4-hydroxy-3-methoxyphenyl)-2-(hydroxymethyl)-2,3-dihydro-1,4-benzodioxin-6-yl]-2,3-dihydrochromen-4-one (silibinin A), and (2*R*,3*R*)-3,5,7-trihydroxy-2-[(2*S*,3*S*)-3-(4-hydroxy-3-methoxyphenyl)-2-(hydroxymethyl)-2,3-dihydro-1,4-benzodioxin-6-yl]-2,3-dihydrochromen-4-one (silibinin B), were purchased from Sigma-Aldrich (St. Louis, MO, USA), and the solvents used were of high-performance liquid chromatography (HPLC) grade.

### 4.2. Plants and Extraction

The plants were identified as *C. japonicum* DC. var. *australe* Kitam. by Yen-Hsueh Tseng, collected by Chih-Yi Chang from Chiayi, and cultivated by Jeng-Chuann Yang in nurseries at Taiwan Forestry Research Institute. Plants were divided into flowers, leaves, stems, and roots, which were rinsed, and dried at 50 °C until their weight was stabilized, with no variations exceeding 0.01 g. Subsequently, the dried samples were powdered and extracted using methanol (MeOH) according to previous studies [10,11], and the crude extracts were concentrated under vacuum until the residue did not differ by more than 0.01 g. The residue of crude extract was then suspended in dimethyl sulfoxide (DMSO).

### 4.3. Identification and Quantification of Active Components in Methanol Extracts

For the calibration curves, all chemical standards were dissolved in DMSO and analyzed using binary gradient elution with mobile phase A (95% water, 5% acetonitrile, and 0.1% formic acid) and B (20% water, 80% methanol, and 0.1% formic acid). The gradient started with 30% of B at 0 min, followed by 60% B at 12 min, 60% B at 13 min, and 30% B at 14 min until 16.5 min, using an HPLC system (Shimadzu, Japan) with a monolithic Chromolith RP-C18 column (100 × 3 mm). The signals were acquired using a photodiode array (PDA) and extracted at 285 nm. The injection volume was 2 µL.

### 4.4. C. elegans and Oxidative Stress Assay

This study used wild-type N2 strain *C. elegans* acquired from the *Caenorhabditis* Genetics Center. We followed the standard protocol, maintaining the *C. elegans* at 20 °C to obtain synchronized L1 larvae. The oxidative stress assay was performed as described by [43]. Briefly, L1 larvae were treated with crude extracts, compounds, or 0.1% DMSO (solvent control), which could be safely used for drug delivery in *C. elegans* [44], for 72 h, and then washed three times with M9 buffer. Next, adult worms were randomly selected and exposed to 250 μM juglone for 3.5 h before their survival was scored. The assay was performed in at least 3 biological trials with at least 60 worms in each trial.

### 4.5. Data Analysis

Data were presented as the mean ± SD from at least three independent biological replicates, and one-way analysis of variance with Tukey’s post hoc test using SPSS 22.0 (IBM, Inc., New York, NY, USA) to compare any differences between groups. An asterisk (*p* < 0.05), two asterisks (*p* < 0.01), and n.s. (*p* > 0.05) are used to indicate statistical analysis results.

## 5. Conclusions

In summary, in this study we conducted a comprehensive investigation into the antioxidant potential of different parts of the *Cirsium japonicum* DC. var. *australe* Kitam. plant, including the flowers, leaves, stem, and roots. We observed variations in the dry weight across the different plant parts. While the yields of crude extracts from flowers, leaves, and roots exhibited similarities, the stem yielded the smallest amount. Additionally, leaves demonstrated the highest crude methanol extract yield, followed by the stem, roots, and flowers. Our results demonstrated that 10 mg/L of flower, leaf, and stem extracts significantly increased the survival of *C. elegans* under juglone-induced oxidative stress. We further conducted chromatographic analysis and successfully separated key compounds, including taxifolin, silychristin, silydianin, silibinin A, and silibinin B. We found that taxifolin and silychristin existed across all plant parts of *C. japonicum* DC. var. *australe* Kitam., whereas silydianin was exclusive to the leaf, stem, and root extracts. Silibinin A and B were absent in the crude methanol extracts of the *Cirsium* plant. Taxifolin, at concentrations of 1 mg/L and 10 mg/L, significantly increased the survival of *C. elegans* while silychristin exhibited antioxidative effects at the lowest observed effect level of 0.1 mg/L. Silydianin did not demonstrate significant antioxidative activity at the tested concentrations. Taken together, these findings indicate that taxifolin and silychristin are pivotal compounds responsible for the antioxidative activity of the crude methanol extracts from *C. japonicum* DC. var. *australe* Kitam. in *C. elegans*. These results offer insights into the potential applications of the *Cirsium* plant in the fields of health and medicine.
